# Germ Cell Transplantation Using Sexually Competent Fish: An Approach for Rapid Propagation of Endangered and Valuable Germlines

**DOI:** 10.1371/journal.pone.0006132

**Published:** 2009-07-02

**Authors:** Sullip K. Majhi, Ricardo S. Hattori, Masashi Yokota, Seiichi Watanabe, Carlos A. Strüssmann

**Affiliations:** Department of Marine Bioscience, Tokyo University of Marine Science and Technology, Tokyo, Japan; Temasek Life Sciences Laboratory, Singapore

## Abstract

The transplantation of germ cells into adult recipient gonads is a tool with wide applications in animal breeding and conservation of valuable and/or endangered species; it also provides a means for basic studies involving germ cell (GC) proliferation and differentiation. Here we describe the establishment of a working model for xenogeneic germ cell transplantation (GCT) in sexually competent fish. Spermatogonial cells isolated from juveniles of one species, the pejerrey *Odontesthes bonariensis* (Atherinopsidae), were surgically transplanted into the gonads of sexually mature Patagonian pejerrey *O. hatcheri*, which have been partially depleted of endogenous GCs by a combination of Busulfan (40 mg/kg) and high water temperature (25°C) treatments. The observation of the donor cells' behavior showed that transplanted spermatogonial cells were able to recolonize the recipients' gonads and resume spermatogenesis within 6 months from the GCT. The presence of donor-derived gametes was confirmed by PCR in 20% of the surrogate *O. hatcheri* fathers at 6 months and crosses with *O. bonariensis* mothers produced hybrids and pure *O. bonariensis*, with donor-derived germline transmission rates of 1.2–13.3%. These findings indicate that transplantation of spermatogonial cells into sexually competent fish can shorten considerably the production time of donor-derived gametes and offspring and could play a vital role in germline conservation and propagation of valued and/or endangered fish species.

## Introduction

Germ cell (GC) transplantation (GCT) is a powerful reproductive technique pioneered by Brinster and colleagues in 1994 [1]. It consists on the transplantation of donor germ cells into the gonads of a surrogate animal for rapid and theoretically unlimited production of gametes from the donor. Over the years, the technique has gained new scientific interest due to the enormous potential for application in reproductive medicine, preservation of valuable and endangered genetic resources, and animal reproduction [1]–[3]. Furthermore, GCT also has implications for understanding the regulation of germ cell development and stem cell biology [1].

The first attempts to perform GCT in fish were performed in rainbow trout and consisted in the grafting of pieces of testes into isogeneic animals, resulting in donor-derived spermatogenesis [4], [5]. These studies were followed by the development of novel approaches based on the transplantation of primordial germ cells (PGCs) into the celomic cavity of fish hatchlings and/or the blastodisc of embryos (and the ability of the PGCs to migrate towards and find their way into the germinal ridges) [6], [7]. More recently, Lacerda et al. [8] and Takeuchi et al. [9], [10] demonstrated that GCT in fish, as originally devised in mammals [1], [3], does not need to be performed with PGCs as transplantation of syngeneic or xenogeneic spermatogonia also resulted in colonization of the recipient gonads by the transplanted cells [8], [11]. Furthermore, when these cells were transplanted to rainbow trout hatchlings, they resulted in donor-derived functional gametes that were capable of fertilization and generation of viable offspring [11]. These results confirm the technical feasibility and the great potential of GCT. However, GCT using fish embryos and/or hatchlings as recipients requires sophisticated instruments and skills for cell transplantation into the blastodisc of embryos or the peritoneal cavity of the small, sometimes only a few millimeters long larvae. In addition, the animals transplanted at these early stages can take a considerably long time to reach adulthood and to produce donor-derived functional gametes, adding considerably to the cost of production of offspring from surrogate parents and hindering commercial application in hatcheries. In contrast, development of surrogate broodstock involving transplantation of gonial cells (spermatogonia or oogonia) into sexually mature hosts which have been naturally or experimentally depleted of endogenous germ cells [8], [12] appears to be suited for almost immediate production of donor-derived gametes. This possibility has been experimentally demonstrated in mammals [1], [13], but it has never been tested in adult fish beyond cell transplantation and demonstration of colonization of the recipient gonads [8].

To test the feasibility of this approach in fish, we performed intra-gonadal surgical GCT between two congeneric atherinopsid fishes as models of donor and recipient. Here we report that transplantation of spermatogonia from pejerrey *Odontesthes bonariensis* into Patagonian pejerrey *O. hatcheri*, previously depleted of endogenous germ cells by Busulfan and high water temperature treatments, resulted in recolonization of recipient testes. The transplanted cells successfully developed into functional sperm and were used in artificial fertilization with eggs of the donor species, generating viable progeny of donor origin. These results demonstrate the viability of xenogeneic GCT into sexually mature fish up to the generation of viable offspring.

## Results

### Colonization of Recipient Gonads by Donor GCs

**Figure 1:**
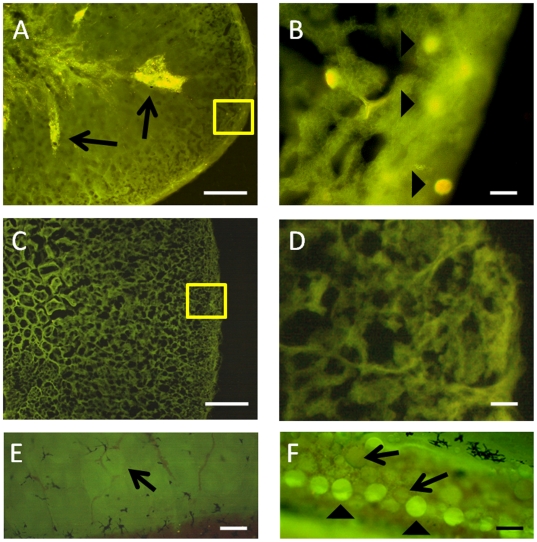
Distribution of CFDA-SE-labeled (2 µM/10 min RT) donor germ cells in recipient gonads 4–6 weeks after transplantation. A,B) Four weeks after transplantation, the donor germ cells were found distributed throughout the seminiferous lobules (arrows) and a small number, presumably spermatogonia, have reached the blind end of the seminiferous lobules (arrowheads; B is a high magnification of the box in A). C,D) Corresponding control sections from non-transplanted gonads (D is a high magnification of the box in C) showing the absence of fluorescent cells. E) Whole-mount preparation of a non-transplanted control gonad showing a cyst of spermatogonia near the blind end of the seminiferous lobule (arrow). F) Whole-mount preparation of a gonad from a transplanted animal six weeks after transplantation showing that donor-derived spermatogonia (arrowheads) have undergone proliferation along with endogenous (arrows) cells in the blind end of the seminiferous lobules of recipient gonads. Scale bars indicate 100 µm (A, C) and 20 µm (B, D, E and F).

The presence of donor-derived GCs in recipient gonads, as opposed to control (non-transplanted) gonads, was confirmed by tracking CFDA-SE (Carboxyfluorescein diacetate, succinimidyl ester)-labeled cells (Figure 1A–F). The process of colonization of the recipient gonads by donor GCs could be broadly divided into two phases. First, during the initial weeks, transplanted cells were randomly distributed throughout the seminiferous lobules (Figure 1A) with only a small number reaching the blind end of the lobule (cortical region of the testis; Figure 1B). This initial step of colonization, namely the settlement of stem spermatogonia in the cortical region of the testis [14], was evident in 2 out of the 3 animals observed 4 weeks after transplantation. In the second phase, donor cells in the blind end of the lobules proliferated and formed a monolayer network along the cortical region of seminiferous lobules (Figure 1F). This stage was observed in 1 out of 3 animals observed 6 weeks after transplantation.

### Spermatogenesis by Transplanted Cells and Detection of Donor-Derived Sperm

**Figure 2:**
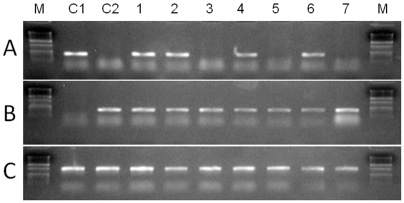
PCR analysis of sperm from 7 recipients eight months after the germ cell transplantation. The primers used were an *O. bonariensis*-specific sequence (A), an *O. hatcheri*-specific sequence (B) and β-actin (C) as a template control. Control lanes include a pure *O. bonariensis* (C1) and an *O. hatcheri* (C2). Donor-derived (*O. bonariensis*) spermatozoa were detected in the sperm of four recipients shown in lanes 1, 2, 4 and 6.

**Figure 3:**
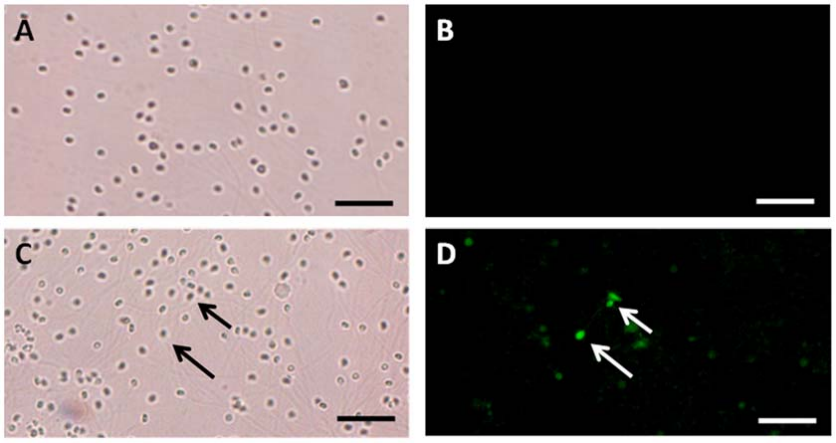
Visualization of donor-derived spermatozoa in sperm collected from surrogate fathers 10 months after transplantation. A) Bright field view of non-transplanted control spermatozoa. B) Corresponding view of A) under fluorescent light. C) Bright field view of sperm from a surrogate father. D) Corresponding view of C) under fluorescent light showing fluorescent (CFDA-SE-labeled) donor-derived cells (arrows). Scale bars indicate 20 µm.

Both the transplanted and the age-matched, non-transplanted males that were experimentally depleted of endogenous germ cells had low milt density at the beginning but the amount of sperm produced increased with time (see Figure S1). Recovery of sperm density was faster in non-transplanted than in GC-transplanted animals but both groups had similar values after 12 months. PCR testing of the sperm at 6 and 8 months after transplantation revealed the presence of *O. bonariensis* (donor)-derived spermatozoa in 4 out of 20 (20%) of the GC-transplanted recipients (Figure 2) and donor-derived spermatozoa were visually identified in the milt of these recipients by the presence of the fluorescent label (Figure 3A–D).

### Generation of Donor-Derived Offspring from Surrogate Fathers

**Figure 4:**
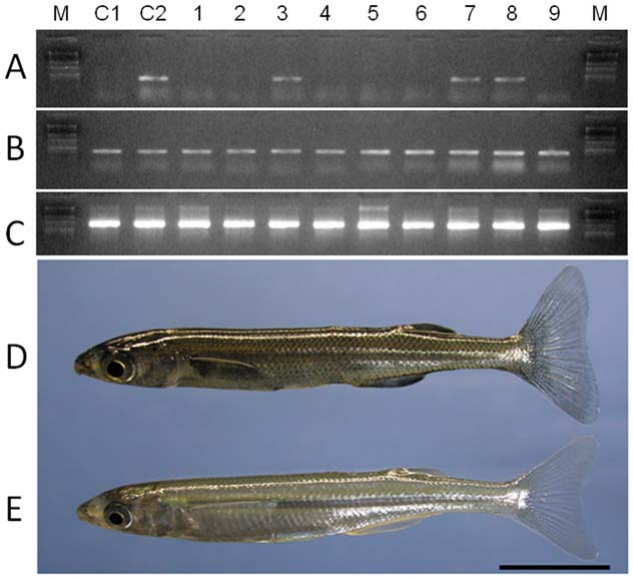
PCR analysis of 9 individual offspring from the cross between a surrogate *O. hatcheri* father (#2) and an *O. bonariensis* mother. The primers used were an *O. hatcheri*-specific sequence (A), an *O. bonariensis*-specific sequence (B) and β-actin (C) as a template control. Control lanes include a pure *O. bonariensis* (C1) and an artificially produced hybrid between an *O. hatcheri* male and an *O. bonariensis* female (C2). Individuals shown in lanes 3, 7, and 8 are hybrids of the two species (D) and those shown in lanes 1, 2, 4–6, and 9 are pure *O. bonariensis* (E). Scale bar indicates 1 cm (D and E).

These four recipients with *O. bonariensis*-derived spermatozoa were subsequently used in progeny testing by artificial insemination with batches of eggs from *O. bonariensis* mothers. The crosses produced viable offspring with normal fertilization and hatching rates and which were estimated by PCR analysis to contain 1.2–13.3% pure *O. bonariensis* in addition to hybrids between the two species (Table 1, Figure 4), suggesting that donor-derived germ cells could differentiate into fully functional spermatozoa in the xenogeneic recipients.

**Table 1:** Results of artificial insemination of *O. bonariensis* eggs with sperm derived from surrogate *O. hatcheri* fathers transplanted with *O. bonariensis* (donor) germ cells.

Surrogate fathers*	Number of eggs (n)	Fertilization (%) (n)	Hatching (%) (n)	Donor-derived germline transmission (%) (n)
#1	105	90.5 (95)	63.2 (60)	8.3 (5)
#2	75	100.0 (75)	80.0 (60)	13.3 (8)
#3	96	93.7 (90)	88.8 (80)	1.2 (1)
#4	100	95.0 (95)	97.9 (93)	3.2 (3)
Control	175	100.0 (175)	85.7 (150)	NA

* PCR-positive recipients that produced donor-derived gametes 6 months after the GCT.

## Discussion

In the present study, donor germ cells harvested from sexually immature *O. bonariensis* testes were successfully transplanted into partially sterilized testes of sexually competent *O. hatcheri* by surgical intervention. As observed in mammals [3], [15]–[17], transplantation resulted in the recolonization of the seminiferous epithelium, resumption of spermatogenesis, and production of functional spermatozoa. Therefore, we conclude 1) that repeated administration of Busulfan and rearing at a high temperature cause pronounced germ cell loss, 2) that testes treated in this way remain sexually competent and able to support gametogenesis, as already noted previously [8], [12], [18], [19], and 3) that GCT into these gonads using simple injection techniques results in recolonization of the recipient gonads with the transplanted germ cell. The simple but effective procedures demonstrated herein could have immediate applicability in hatcheries and other seed production facilities, with great potential for the production of valuable species and the conservation (or restoration) of endangered species.

Regardless of the differences in testicular structure between our fish model (Pisces, Atheriniformes; “unrestricted lobular” testis whereby spermatogonia are restricted to the blind end of the seminiferous lobules), other fish species and mammals (unrestricted lobular and anastomosing tubular testes, respectively, whereby spermatogonia are found along the lobules or tubules) [20], the process of recolonization of the recipient *O. hatcheri* gonads by the transplanted GCs showed many similarities to that reported in GC-transplanted tilapia [8] and mice [21]. For instance, there was considerable interlobular variability in CFDA-SE-positive cell distribution during the first weeks after transfer but many labeled cells eventually settled along the blind end of the seminiferous lobules and began proliferating within weeks of transplantation. Assuming that only spermatogonial stem cells (type A spermatogonia) have the capability to migrate and settle at the basement membrane and resume the process of spermatogenesis [14], we may surmise that these cells were spermatogonial stem cells. This conclusion is also borne out by the fact that the recipients have been repeatedly stripped of sperm but continue to produce donor-derived spermatozoa to this date (more than 14 months after the GCT; results not shown). This is an indication that the transplanted cell population included cells with the potential for self-renewal and proliferation. Type A spermatogonia are able to undergo regular self-renewing divisions and maintain a pool of undifferentiated germ cells, as opposed to the type B spermatogonia that divide and go on to differentiate into spermatocytes, spermatids, and finally spermatozoa [22]–[23]. It is also noteworthy that the injected cells were able to migrate and settle at the germinal epithelium amidst the endogenous GCs. It has been reported that transmembrane protein molecules present at the junctional complex located in the seminiferous tubules transduce signals, maintain cell polarity, and mediate germ cell migration [24]. Although we did not examine the pathways involved in the migration of donor GCs inside the recipient gonads, the results obtained in the present study indicate that the *O. bonariensis* (donor) GCs might sense and respond to molecules released from the blind end of the seminiferous lobules in *O. hatcheri* (recipient). A series of time-course observations prior to and after the 4–6 week post-GCT period, which was the only period observed in this study, may provide a more comprehensive view of the process of gonadal recolonization by donor-derived germ cell populations.

We observed that donor-derived spermatozoa from surrogate fathers had similar functional properties as those of control ones in terms of fertilization and hatching rates, and we could not detect any evidence of defective spermatogenesis. Yet, there have been cases of defective transcription of genetic information by donor cells during intertaxa GCT [25], which seem to appear most prominently when the phylogenetic distance between the donor and recipient is large. In this study we used a congeneric fish model and the transplanted cells were probably immunologically compatible with the recipient's gonadal environment. Nevertheless, 16 of the 20 GCT recipients ultimately did not develop donor-derived spermatozoa and the efficiency of recolonization of the recipient testes by donor cells decreased from approximately 2/3 of the individuals at 4 weeks, to 1/3 at 6 weeks, and finally 1/5 at >6 months, although not thereafter. Thus, we can not rule out entirely the possibility of immunological rejection and resorption of the transplanted cells. On the other hand, the variability observed at 4 weeks (e.g. animals with and without donor cells) might have originated, in part, from leakage of cell suspension from the seminiferous lobules at the time of injection. The variability in the following weeks and months may have been caused by differences in the number of spermatogonial stem cells actually transferred into each recipient, which could not be ascertained in this study. Unfortunately, there are no specific biochemical or morphological markers available to identify spermatogonial stem cells from other germ cell types prior to transplantation. The number of germinal cell colonies recovered from seminiferous tubules in transplanted males has been used as a proxy for the number of spermatogonial stem cells transferred into each recipient [26], but this strategy may not be a precise or timely tool for stem cell quantification, particularly in cases such as ours when the recipients are not completely sterile. Therefore, the development of genetic markers for stem cell identification might constitute a valuable tool for improving the efficiency of GCT.

We recorded a donor-derived germline transmission rate in the progeny ranging between 1.2 to 13.3%. In comparison, when primordial germ cells (PGCs) were transplanted into recipient embryos, their contribution to the germline was reported to be between 2–4% [6]. In this context, our findings seem to support the view that transplanted spermatogonial cells have improved access to the germinal epithelium, where they are able to resume spermatogenesis, if the recipients have been severely depleted of their own GCs prior to GCT [8], [27]. This may be particularly true with sexually competent (mature) animals as in this case. Indeed, transplantation into embryos neutered by either triploidy [10], [11] or an antisense dead end morpholino oligonucleotide [7] originated 100% donor-derived gametes. The proposed use of triploid sterile recipients, whose germ cells are unable to develop into fertile gametes [28]; but see also opposite views [29], may seem to obviate the need for endogenous germ cell depletion [10]. However, the long time required for producing and raising triploid animals to sexual maturity, and the need to develop the techniques for triploid induction in a species phylogenetically close to the target one may limit considerably the applicability of this approach for rare, endangered species. In this regard, the timely production of completely sterile fish, preferably by non-chemical means such as with the use of high temperature [8], [12], [18], [30], could become a useful strategy to save endangered species in situations when time to perform GCT has become a constraint. Further efforts should be devoted to the development of timely methods for complete sterilization of adult, sexually competent GCT recipients.

In conclusion, the most striking result of our study is the production of pure donor-derived progeny (up to 13.3%) in the span of months when donor germ cells were transplanted into the testes of sexually competent male recipients. This approach offers a workable alternative to PGC or spermatogonia transplantation using embryos and/or young hatchlings, which requires sophisticated equipment, skills, and time to yield donor-derived gametes. It offers a way to preserve and propagate the germplasm from animals that are still sexually immature or that have become senile, which therefore can not provide sperm for cryopreservation, and this may be extremely important in the case of rare, endangered species. Ongoing studies are testing (low-tech) refinements in the proposed approach, such as non-surgical transplantation [8], [31] and the suitability of GCT for generation of female gametes, for which cryopreservation techniques have not yet been developed. These developments will make GCT very useful for the timely rescue of fish species facing imminent extinction.

## Materials and Methods

### Experimental Animals and General Rearing Protocols

**Figure 5:**
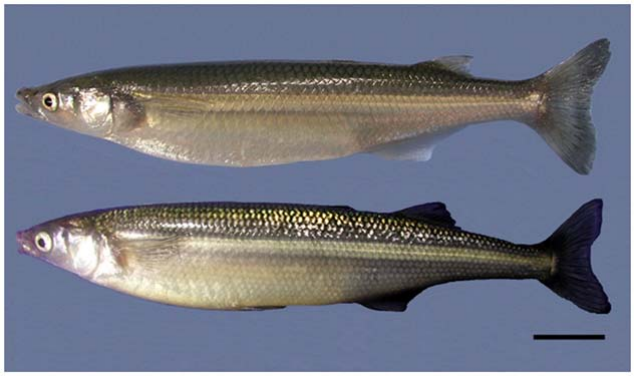
The model fish species used in the study: pejerrey *Odontesthes bonariensis* (donor; top) and Patagonian pejerrey *O. hatcheri* (recipient; bottom). Scale bar indicates 4 cm.

In this study, we used two congeneric species of atherinopsid fishes, the pejerrey *Odontesthes bonariensis* and the Patagonian pejerrey *O. hatcheri* as donor and recipient, respectively (Figure 5). These two species are originally from South America and have been introduced to Japan and other countries as candidates for aquaculture [32]. They can be easily bred in captivity and produce viable hybrid offspring, which can be readily distinguished using genetic markers [33]. For recipient preparation, one year old sexually mature (mean body weight±SD of 37.3±16.8 g) male *O. hatcheri* were procured from the Field Science Center, Tokyo University of Marine Science and Technology, Yoshida Station. Fish were stocked in 200 L tanks at a density of 7.5 kg of fish per m^3^ and reared in flowing brackish water (0.2–0.5% NaCl) under a constant light cycle (15L9D). The fish were acclimated for two weeks at 17°C prior to the treatments for depletion of endogenous germ cells. The *O. bonariensis* used as GC donors were 4–5 months old juveniles and were produced at the rearing facilities of the Tokyo University of Marine Science and Technology at Shinagawa Campus. Both groups of animals were fed pelleted commercial diet four times per day to satiation.

### Recipient Preparation

The endogenous GCs of *O. hatcheri* intended as recipients were depleted according to the procedure described by Majhi et al. [12]. Briefly, fish were reared at a relatively high temperature (25°C) and injected intraperitoneally twice with 40 mg/kg body weight Busulfan (Sigma Aldrich, St. Louis, MO, USA) at a 4 week interval. Histological observation (n = 5) confirmed that treated animals were severely depleted of endogenous GCs 4 weeks after the second injection of Busulfan (see Figure S2). Recipients were then used in GCT within 3 days of termination of the GC depletion treatment.

### Isolation and Labeling of Donor Cells

Donor males were sacrificed by anesthetic overdose and the testes were excised and rinsed in phosphate-buffered saline (PBS; pH = 8.2). The testicular tissue was finely minced and incubated in a dissociating solution containing 0.5% Trypsin (pH 8.2; Worthington Biochemical Corp., Lakewood, NJ), 5% Fetal Bovine Serum (JRH Biosciences, Lenexa, KS), and 1 mM Ca^2+^ in PBS (pH 8.2) for 2 hr at 22°C. The dispersed testicular cells were sieved through a nylon screen (mesh size 50 µm) to eliminate the non-dissociated cell clumps. Cells were then suspended in a discontinuous Percoll (Sigma-Aldrich, St.Louis, MO, USA) gradient of 50%, 25% and 12%, which was prepared by dilution of the stock solution with PBS (pH = 8.2), and centrifuged at 200×*g* for 20 min at 20°C. The phase containing the largest cells (presumably spermatogonia [18]) was harvested, the cells were rinsed, and then subjected to a cell viability test by the trypan blue (0.4% w/v) exclusion assay. The cells were then exposed to the CFDA-SE Cell Linker (Invitrogen Life Technology, Carlsbad, CA, USA) at a concentration of 2 µM (room temperature, 10 minutes) to label the cells for tracking their behavior inside the recipient gonads. The staining procedure was stopped by addition of an equal volume of heat-inactivated fetal bovine serum. Labeled cells were rinsed three times to remove unincorporated dye, suspended in Dulbecco Modified Eagle Medium (Life Technologies, Rockville, MD) with 10% fetal bovine serum, and stored on ice until transplantation.

### Cell Transplantation Procedures

Thirty one recipients were used for surgical GCT. Surgical intervention was used because it allows direct delivery and visualization of the donor cells into the recipient gonad. For this purpose, the fish were anesthetized in 200 ppm phenoxyethanol (Wako Pure Chemicals Ind., Osaka, Japan) and placed on an operation platform under a microscope where they received a constant flow of oxygenated, cool water containing 100 ppm of the anesthetic through the gills. To prevent desiccation, the surface of the fish was moisturized during the entire GCT procedure, which took about 20 min per fish on average. An approximately 1.5 cm-long midline incision was made in abdomen and the gonads were carefully lifted from the coelomic cavity and hold in place with the help of a sterile soft plastic spatula. A micro syringe and fine glass needle were used to inject the cell suspension into the medullar region of each testicular lobe. Each lobe of the testis was injected with 50 µl of cell suspension containing approximately 8×10^2^ cells/µl, at a flow rate of approximately 10 µl/min. Trypan blue was added to the injection medium prior to transplantation to allow visualization of the cell suspension inside the needle and inside the gonad after injection. Repeated reentry and readjustment of the needle position were avoided to minimize tissue damage and leakage of cells. The abdominal incision was stitched with nylon surgical thread and topically treated with 10% Isodine (Meijiseika Ltd., Tokyo, Japan), and the fish were resuscitated in clean water.

### Post-Transplantation Analysis of the Fate of Donor Cells

Post-transplantation analysis of the fate of donor cells was performed first by fluorescent microscopy at 4 and 6 weeks after injection. For this purpose, the testes from three animals chosen randomly on each sampling were removed, washed in PBS (pH 8.2), macroscopically observed for the degree of dispersion of the cell suspension (see Figure S3), and examined under a fluorescent microscope (Nikon Eclipse E600, Tokyo, Japan) for the presence of CFDA-SE-positive donor cells. Samples were then frozen in liquid nitrogen and sectioned to a thickness of 10 µm using a Leica CM 1500 (Germany) cryostat. Sections from representative portions of these testes were observed with the fluorescent microscope for examination of the distribution of donor GCs.

The fate of the donor cells was then examined by sperm density and molecular (PCR) analysis between 6 and 12 months after transplantation. On each occasion, 10–30 µl of sperm was collected from each of 20 transplanted males and 5 control males. Sperm was manually stripped by gentle abdominal pressure after careful removal of urine and wiping of the genital papilla. Ten microliters of sperm were then diluted 1,000 times with PBS and the density of spermatozoa was counted in a hemocytometer (Kayagaki Irika Kogyo Co., Ltd., Tokyo, Japan) under a microscope. Some of the spreads of sperm used for counting were also observed under the fluorescent microscope for the detection of CFDA-SE labeled donor cells. Sperm samples collected at 6 and 8 months were subjected to PCR analysis for molecular detection of donor-derived cells. DNA was extracted by the standard phenol:chloroform protocol and subjected to PCR analysis with *O. bonariensis*-specific primers (forward 5′-CAG TGC AGG TCC AGC ATG GG-3′ and reverse 5′-TGT TCC GCC TCA GTG CTT CAG-3′; amplicon size 386 bp) that were designed based on the sequence of the first intron of the *amh* gene from this species (GenBank accession number #FJ977837) using Genetyx Ver 8.2.1 (Genetyx Corp. Tokyo, Japan). Primers for β-actin were used as positive controls (forward: 5′-CTC TGG TCG TAC CAC TGG TAT CG-3′; reverse: 5′-GCA GAG CGT AGC CTT CAT AGA TG-3′). The PCR reactions were run in a Mastercycler EP Gradient S (Eppendorf, Hamburg, Germany) and consisted of an initial denaturation at 94°C for 3 min, 30 cycles of 94°C for 30 sec, 70°C for 30 sec and 72°C for 1 min, followed by elongation at 72°C for 5 min. PCR products were electrophoresed on an agarose gel (1%), stained with ethidium bromide, and photographed for later analysis.

### Artificial Insemination and Progeny Analysis

Once the sperm density of transplanted animals have returned to pre-treatment levels and the PCR analysis showed the presence of donor-derived sperm (e.g. after 6 months), we performed artificial insemination using the PCR-positive sperm to assess the viability of the *O. bonariensis* spermatozoa produced by the surrogate *O. hatcheri* fathers. About 10 µl of sperm from each of these males was used to fertilize a batch of *O. bonariensis* eggs, which were then incubated under flowing brackish water at 20°C until hatching. The larvae from each cross were reared separately until sampling for PCR analysis of their genetic background at 8–10 days after hatching. The template DNA was extracted from each sample and subjected to PCR analysis using recipient's (*O. hatcheri*) specific primers (forward 5′-ATG ATC AGC AGC TGA GCC CAC CTC C-3′ and reverse5′-TGT TCC GCC TCA GTG CTT CAG-3′; amplicon size 386 bp) also designed based on the sequence of the first intron of the *amh* gene from this species (GenBank accession number #FJ977836) using Genetyx Ver 8.2.1 (Genetyx Corp. Tokyo, Japan). The PCR conditions were the same as described above. The genetic information of each individual was used to calculate the donor germline transmission rates as described elsewhere [11].

### Statistical Analysis

The statistical significance of the differences in sperm production between groups was analyzed by one-way analysis of variance (ANOVA) followed by the Tukey's multiple comparison test using Graphpad Prism ver. 4.00 (Graphpad Software, San Diego, Carlifornia, USA). Data are presented as mean±SD and differences between groups were considered as statistically significant at *P*<0.05.

## Supporting Information

Figure S1 Sperm density in germ cell transplanted recipients and non-transplanted (negative control) animals between 6 and 12 months after transplantation. Columns with different letters vary significantly (Tukey's multiple comparison test, P<0.05).
(60.78 MB TIF)

Figure S2 Histological appearance of the testes of Patagonian pejerrey O. hatcheri in control and Busulfan-high temperature treated groups. A,B) Normal testis showing the thick germinal epithelium, the radially-oriented seminiferous lobules, and large cysts of spermatogonia (arrows) in the blind end of the seminiferous lobule (B is a high magnification of the box shown in A). C,D) Testis from the high temperature (25°C) - Busulfan (two injections of 40 mg/kg 4 weeks apart) treatment group at 8 weeks showing virtual lack of spermatogonia (D is a high magnification of the box shown in C). Scale bars indicates 100 µm (A,C) and 20 µm (B,D).
(5.69 MB TIF)

Figure S3 Visualization of the dispersal of the cell suspension through the gonad after transplantation. A) Macroscopic appearance of a control testis. B) Appearance of the testis 4 weeks after germ cell transplantation (note the diffusion of the marker trypan blue through all areas of the testis). Scale bars indicate 1 cm.
(1.30 MB TIF)
