# Supplementary figures and images for: Germ Cell Transplantation Using Sexually Competent Fish: An Approach for Rapid Propagation of Endangered and Valuable Germlines
Source: PLoS One. 2009 Jul 2;4(7):e6132. doi: 10.1371/journal.pone.0006132 (PMC2700270; doi:10.1371/journal.pone.0006132)

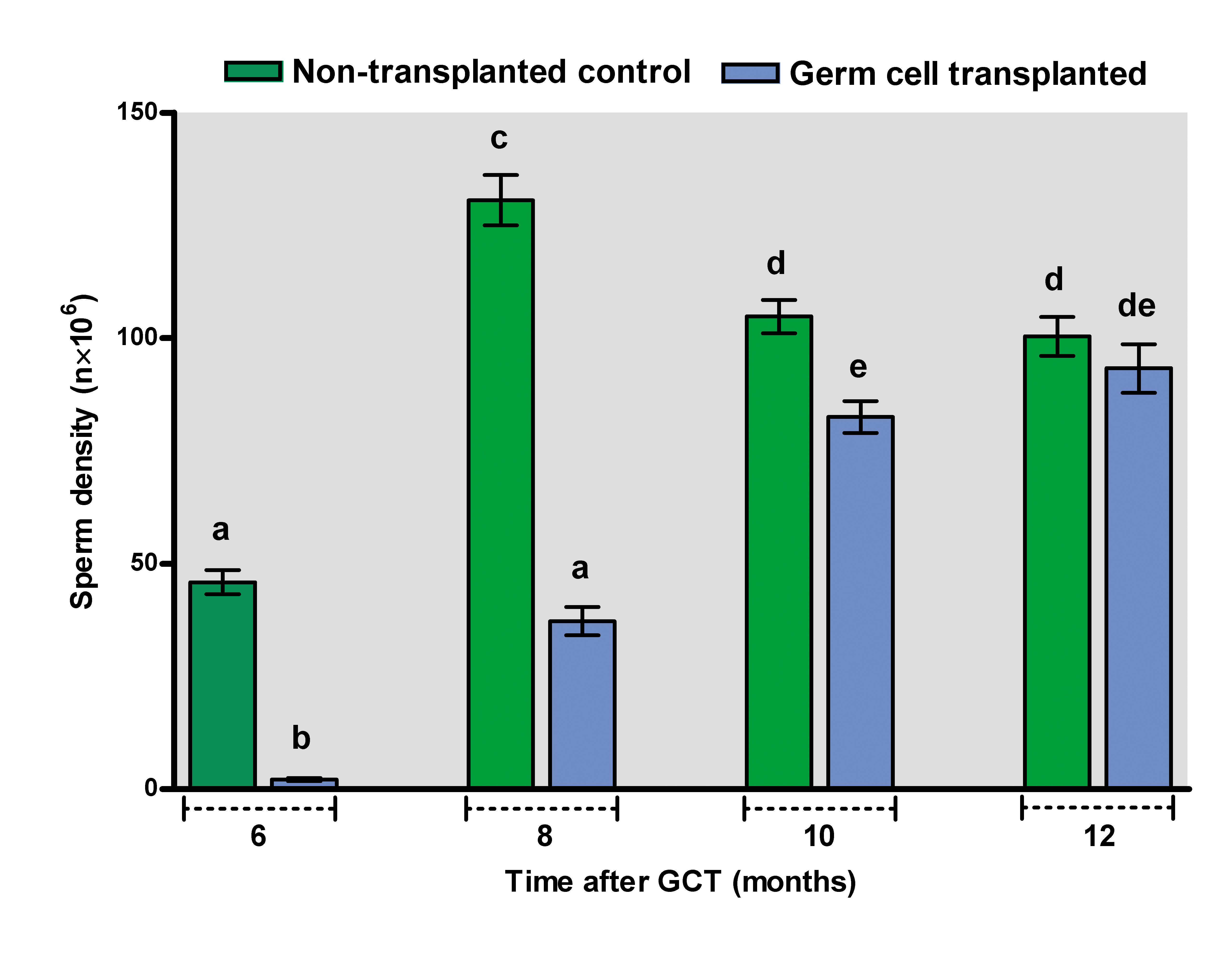

Supplement: Figure S1 — Sperm density in germ cell transplanted recipients and non-transplanted (negative control) animals between 6 and 12 months after transplantation. Columns with different letters vary significantly (Tukey's multiple comparison test, P<0.05). (60.78 MB TIF) [file pone.0006132.s001.tif]

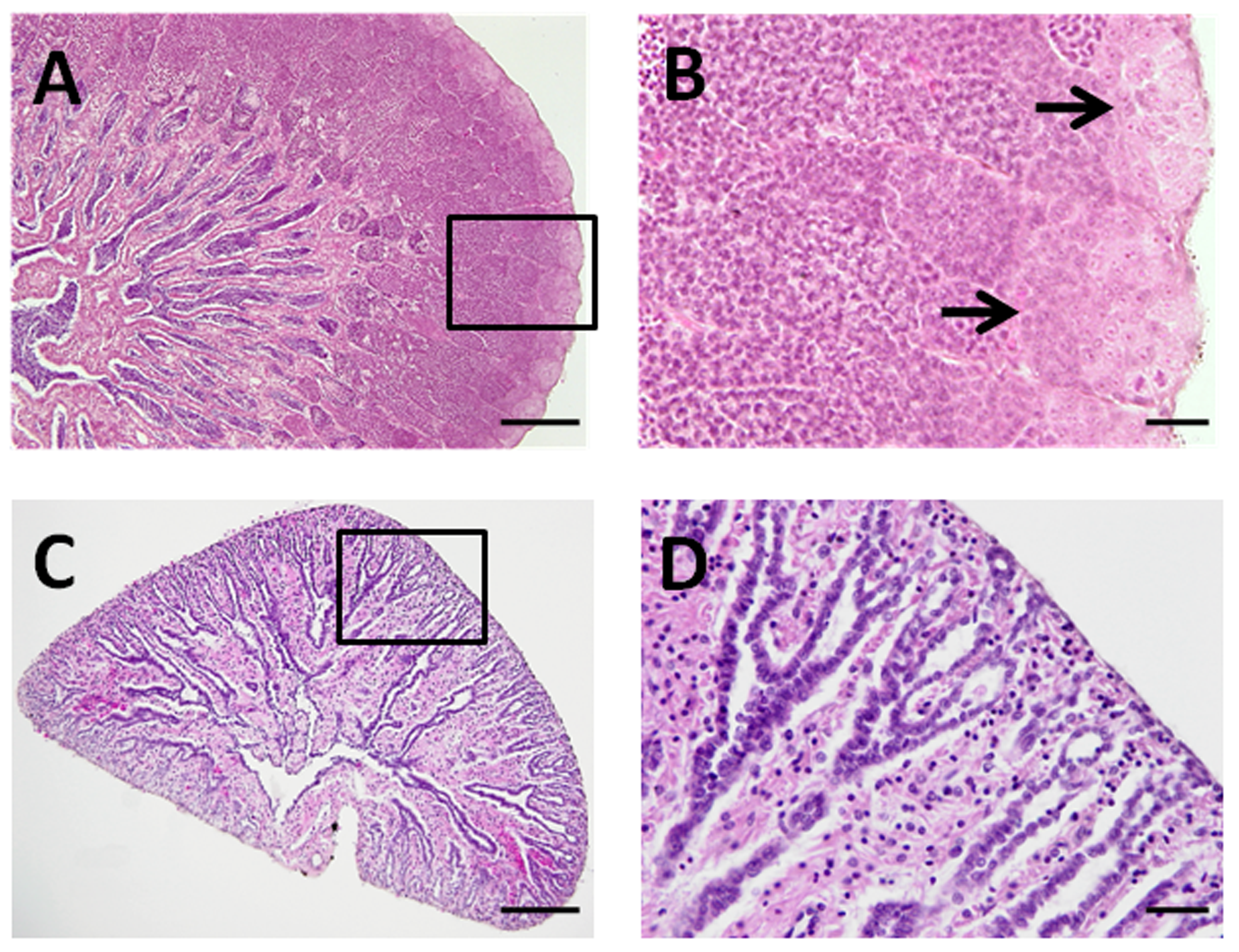

Supplement: Figure S2 — Histological appearance of the testes of Patagonian pejerrey O. hatcheri in control and Busulfan-high temperature treated groups. A,B) Normal testis showing the thick germinal epithelium, the radially-oriented seminiferous lobules, and large cysts of spermatogonia (arrows) in the blind end of the seminiferous lobule (B is a high magnification of the box shown in A). C,D) Testis from the high temperature (25°C) - Busulfan (two injections of 40 mg/kg 4 weeks apart) treatment group at 8 weeks showing virtual lack of spermatogonia (D is a high magnification of the box shown in C). Scale bars indicates 100 µm (A,C) and 20 µm (B,D). (5.69 MB TIF) [file pone.0006132.s002.tif]

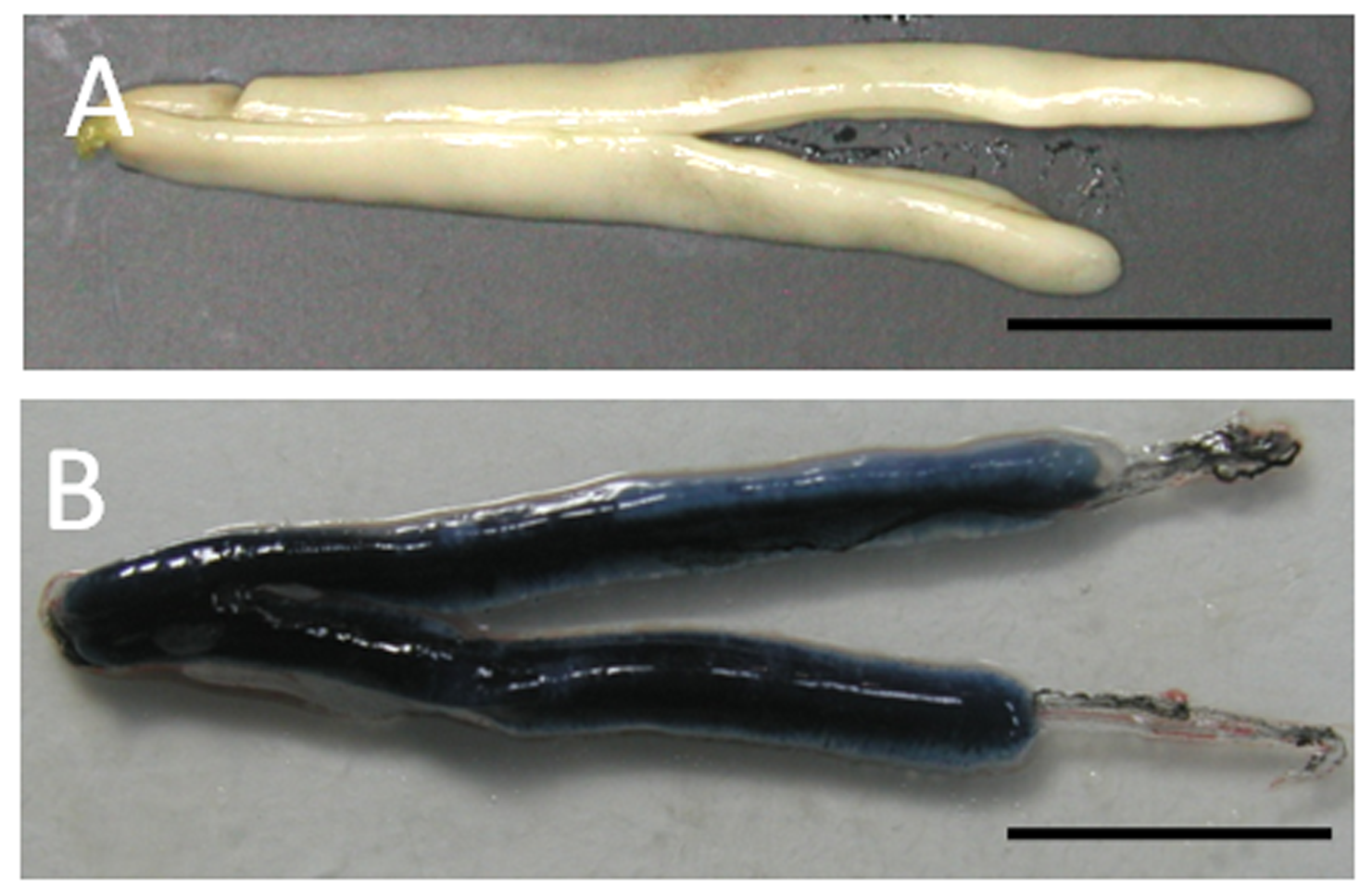

Supplement: Figure S3 — Visualization of the dispersal of the cell suspension through the gonad after transplantation. A) Macroscopic appearance of a control testis. B) Appearance of the testis 4 weeks after germ cell transplantation (note the diffusion of the marker trypan blue through all areas of the testis). Scale bars indicate 1 cm. (1.30 MB TIF) [file pone.0006132.s003.tif]
